# Oregano essential oil *(Origanum vulgare)* induces death in a resistant strain of *Leishmania infantum* isolated from a naturally infected dog through increased production of reactive oxygen species, mitochondrial damage and cell metabolic disruption

**DOI:** 10.3389/fcimb.2025.1601429

**Published:** 2025-06-18

**Authors:** Ana Carolina Jacob Rodrigues, Danielle Lazarin Bidoia, Amanda Cristina Machado Carloto, Virgínia Márcia Concato-Lopes, Mariana Barbosa Detoni, Ellen Mayara Souza Cruz, Sara Mayumi Suzuki, Fabricio Seidy Inoue, Taylon Felipe Silva, Fabiano Borges Figueiredo, Wander Rogério Pavanelli

**Affiliations:** ^1^ Laboratory of Immunoparasitology of Neglected Diseases and Cancer, Department of Immunology, Parasitology, and General Pathology, Center of Biological Sciences, Londrina State University, Londrina, Brazil; ^2^ Laboratory of Cell Biology, Carlos Chagas Institute - Fiocruz, Curitiba, Brazil; ^3^ Laboratory of Pain, Inflammation, Neuropathy, and Cancer, Department of Immunology, Parasitology, and General Pathology, Center of Biological Sciences, Londrina State University, Londrina, PR, Brazil

**Keywords:** leishmaniasis, alternative treatment, cell death mechanisms, mitochondrial damage, reactive oxygen species

## Abstract

Background: Leishmaniasis is a neglected tropical disease caused by protozoa of the *Leishmania* genus, transmitted by phlebotomine sandflies. Clinical manifestations vary depending on the parasite’s species and the host’s immune system, ranging from self-healing skin lesions to lethal visceral diseases. Visceral leishmaniasis (VL), mainly caused by *L. infantum* and *L. donovani*, is a serious public health issue. Conventional treatment is challenging due to toxicity, long duration, and resistance, highlighting the need for alternative therapies. Oregano essential oil (OEO) has biological effects, including antibacterial, antifungal, and antioxidant actions, making it a potential leishmanicidal agent. To evaluate its activity, promastigotes of two *L. infantum* strains were tested: MS (from naturally infected dogs in Brazil) and a reference strain (MCAN/BR/97/p142). The IC50 values were 12.53 µg/mL and 43.61 µg/mL, respectively, while amphotericin B (AmB) showed lower IC50 values (0.1453 µg/mL and 0.2126 µg/mL). OEO treatment increased reactive oxygen species (ROS) production, mitochondrial damage, lipid droplet accumulation, and autophagic vacuoles, indicating intense cellular stress. Additionally, apoptosis markers such as phosphatidylserine exposure and membrane permeabilization were detected. Fluorescence, scanning (SEM), and transmission (TEM) microscopy revealed morphological and ultrastructural alterations, including membrane blebbing, flagellar damage, intracellular content leakage, and mitochondrial swelling. To assess its anti-amastigote effect, THP-1 cells infected with *L. infantum* strains were treated with OEO. The MS strain showed a lower infection rate but a higher parasite load per macrophage. All tested concentrations (25, 50, and 75 μg/mL) reduced both the number of infected macrophages and intracellular amastigotes. Thus, OEO exhibits leishmanicidal activity in both promastigote and amastigote forms of *L. infantum*, inducing metabolic disruption and cell death, even in strains from naturally infected dogs. These findings highlight OEO’s potential as an alternative treatment for VL.

## Graphical Abstract

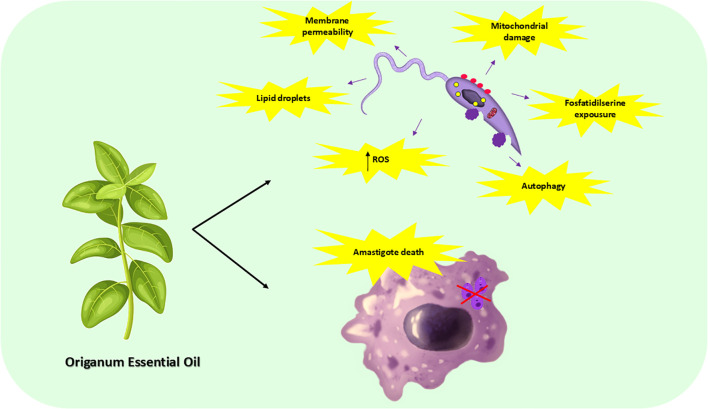

## 1 Introduction

Leishmaniasis is a neglected tropical disease caused by protozoa of the *Leishmania* genus, transmitted through the bite of phlebotomine sandflies (Centers for Disease Control and Prevention (CDC), 2019). Its clinical manifestations vary depending on the parasite species and the host’s immune system, ranging from self-healing skin lesions to lethal visceral disease (Burza et al., 2018).

Visceral leishmaniasis (VL) is an anthropozoonosis, with the domestic dog being its primary reservoir in the Americas. It is mainly caused by the *L. infantum* and *L. donovani* species. VL causes symptoms such as fever, weight loss, splenomegaly which may or may not be associated with hepatomegaly, pallor, and anemia. Among the major infectious- parasitic diseases, VL is considered by the World Health Organization (WHO) a serious public health issue. It is estimated that VL affects about 500.000 people each year, causing over 59,000 deaths, with most cases occurring in Brazil, East Africa, and India (World Health Organization (WHO), 2022).

Currently, the chemotherapy treatment for leishmaniasis primarily relies on drugs such as meglumine antimoniate (Glucantime) and secondary options like amphotericin B (AmB), liposomal AmB, and miltefosine. Yet, sodium stibogluconate (Pentostam), paromomycin, pentamidine and sitamaquine are also available to treat the disease (Majumder et al., 2023). However, conventional treatment is difficult to administer, requires prolonged treatment periods, causes serious adverse effects such as liver and kidney damage, and may lead to the emergence of resistant strains. This highlights the need to explore new compounds with leishmanicidal activity that are safe to the patient (Anversa et al., 2018).

In this sense, there is a growing interest in studying the biological activities of natural compounds, especially essential oils which contain a variety of hydrophobic compounds with antimicrobial potential. The ability of these molecules to diffuse through cell membranes is believed to contribute to the elimination of intracellular parasites, such as *L. tropica, L. major and L. amazonensis* (Bakkali et al., 2008).

In this context, oregano essential oil (OEO) stands out for its biological effects, including its antibacterial, antifungal and antiparasitic actions (Sakkas and Papadopoulou, 2017). The antimicrobial activity of OEO has been attributed to the different chemical classes of compounds, such as terpenes and phenolic compounds, that comprise it. The interaction between these compounds has already been reported, suggesting that the extract as a whole is more effective when compared to its isolated components, such as carvacrol and thymol, the primary constituents of OEO (Bassolé and Juliani, 2012).

In addition, OEO has already demonstrated anti-*Leishmania* action, by inhibiting promastigote and amastigote forms of *L. amazonensis*, and by showing activity in an *in vivo* tegumentary leishmaniasis model (Teles et al., 2019; Tomiotto-Pellissier et al., 2022, 2018). However, to date, there are no studies showing the leishmanicidal potential and mechanisms of action of OEO against isolates of *L. infantum.*

Thus, given the difficulties of current treatment and the parasite’s growing resistance, our aim was to evaluate the activity of OEO against two strains of *L. infantum*: a reference (MCAN/BR/97/p142) and a MS strain; as well as, to elucidate the mechanisms of action and of death involved, and assess its cytotoxicity and activity in infected macrophages. The reference strain analyzed was obtained commercially from cell banks, so has not undergone recent isolation and is maintained in culture. In contrast, the MS strain was isolated from a dog naturally infected with *L. infantum* in Mato Grosso do Sul state, Brazil, which is considered endemic for the disease, predisposing this strain to be more resistant to treatment than the reference strain (Gonçalves et al., 2021).

We were able to infer that the treatment with OEO led to death by apoptosis of the parasite’s promastigotes form, from both commercial and natural sources, as well as significantly reducing the number of infected macrophages and amastigotes per macrophage in both strains tested.

## 2 Materials and methods

### 2.1 Culture of *Leishmania infantum*

The reference strain of *L. infantum* (MCAN/BR/97/p142) was provided by the State University of Maringá and was originally obtained from the ATCC cell bank.

The isolated strain of *L. infantum* (MS) was obtained from a naturally infected female dog of no defined breed, aged approximately 5 years old, from the municipality of Campo Grande, state of Mato Grosso do Sul, Brazil, according to Gonçalves et al. (2021).

Promastigote forms of both strains of *Leishmania infantum* (MCAN/BR/97/p142 and MS) were maintained in culture containing Medium 199, 1% L-glutamine, 10% sodium bicarbonate, 10% FBS, 10 u/mL penicillin, and 10 µg/mL streptomycin (GIBCO, Invitrogen, New York, USA). The parasites were maintained in 75 cm^2^ culture flasks in an B.O.D incubator (24°C).

### 2.2 Oregano essential oil

*Origanum vulgare* essential oil was obtained from Ferquima Indústria e Comércio de Óleos Essenciais (São Paulo, SP, Brazil). This oil was extracted by steam distillation and its density (0.954 g/mL) and composition (main components: carvacrol (72%), thymol (2%), γ- terpinene (4,5%), p-cymene (4%) and linalool (4%)) were described in its technical report (CAS number 84012-24-8, batch 224) (FERQUIMA • Ind. e Com. de Oléos essenciais, 2025).

### 2.3 Anti-promastigote assay

*Leishmania infantum* promastigotes (10^6^) were plated and treated with different concentrations of OEO (25, 50, 100 e 150 μg/mL) for 24 h at 25°C. After this period of treatment, the parasites were counted in a Neubauer chamber. The results were expressed as % of viable promastigotes in relation to the control, which was considered to be 100% viable. *L. infantum* promastigote maintained in culture without any treatment or with vehicle (DMSO 0,2% diluted in Medium 199) were used as negative controls; and amphotericin B (AmB) (1µM) (Sigma-Aldrich, St. Louis, MO, USA) was used as positive control. The half-maximal inhibitory concentration (IC_50_) in parasites was calculated by non-linear regression to the dose–response curve using Graph Pad Prism 6.01 software (GraphPad Software, Inc., USA, v.5).

### 2.4 Determination of reactive oxygen species

To evaluate the ROS generation, promastigote forms of *L. infantum* (10^6^) treated with OEO (IC_50_ and 2× IC_50_) for 24 h were incubated with 5 μM of the 2’,7’-dichlorofluorescein diacetate probe (H2DCFDA) (Sigma, St. Louis, MO) for 45 min at 25°C on a black plate. ROS was measured by the increase in fluorescence caused by the conversion of non-fluorescent dye to highly fluorescent 2’,7’-dichlorofluorescein (DCF), at excitation wavelength of 488 nm and emission of 530 nm in a fluorescence microplate reader (Victor X3, PerkinElmer, Finland). Hydrogen peroxide (H_2_O_2_) was used as the positive control. Additionally, micrographs of OEO-treated promastigotes were acquired using an EVOS ^®^ Microscope FL Auto Cell imaging system (Thermo Fisher, Multiskan GO, Waltham, MA, USA) at 200× magnification. The obtained fluorescence values were normalized by the number of parasites.

### 2.5 Determination of the mitochondrial membrane potential (ΔΨm)

Promastigote forms (10^6^) were treated with OEO (IC_50_ and 2× IC_50_) for 24 h at 25°C. After that, parasites were washed with PBS and incubated with 25 nM of tetramethylrhodamine ethyl ester (TMRE; Sigma-Aldrich, St. Louis, MO) for 30 min at 25°C. The fluorescence was measured in a Victor X3 spectrofluorometer (PerkinElmer) at an excitation wavelength of 480 nm and an emission wavelength of 580 nm. Carbonyl Cyanide m-chlorophenylhydrazone (CCCP) was used as a positive control (100 μM) (Sigma). Additionally, micrographs of OEO**-**treated promastigotes were acquired using an EVOS^®^ Microscope FL Auto Cell imaging system (Thermo Fisher) at 200x magnification.

### 2.6 Detection of lipid droplets

Quantification of LD was performed using the Nile Red (NR) marker. After 24 h of treatment with OEO (IC_50_ and 2×IC_50_), promastigote forms were incubated with NR (0.5 μM) for 30 min at 25°C. Fluorescence was measured in a fluorescence microplate reader (Victor X3, PerkinElmer) at an excitation of 530 nm and emission of 635 nm. PBS without medium was used as the positive control. The obtained fluorescence values were normalized by the number of parasites.

### 2.7 Formation of autophagic vacuoles

The formation of autophagic vacuoles in promastigote forms of *L. infantum* was analysed according to (Machado et al., 2017) with modifications. Promastigote forms (10^6^) treated with OEO (IC_50_ and 2×IC_50_) for 24 h were marked with monodansylcadaverine (MDC; 50 μM) for 1 h at 25°C. The parasites were then analyzed using a fluorescence microplate reader (Victor X3, PerkinElmer) with excitation at 380 nm and emission at 525 nm.2.8 Evaluation of morphological and ultrastructural changes of parasitic cells by electron microscopy

Both promastigote and amastigote forms were examined by electron microscopy. For the promastigote analysis, 1x10^6^ promastigotes were treated for 24 h with the IC_50_ of OEO, and then collected by centrifugation and washed in 0.01 M PBS (pH 7.2). For the microscopy of amastigote forms, THP-1 monocytes (1x10^5^) were first plated, then incubated with PMA 0.5% for differentiation into macrophages; after 48 h, these cells were washed twice with sterile PBS and then maintained in RPMI 1640 medium (10% SFB). Next, 1x10^6^ promastigotes of *L. infantum* were added to the THP-1 cells for 24 h, and then were treated with the IC_50_ of OEO for 24 h.

Scanning electron microscopy (SEM) was used to assess morphological changes of the parasite. The parasites were fixed with 2.5% glutaraldehyde in 0.1 M sodium cacodylate buffer followed by washing with 0.1 M sodium cacodylate buffer. The parasites and infected cells were covered with a layer of poly-L-lysine solution. The adhered cells were next washed with 0.1 M sodium cacodylate buffer, post-fixed in 1% osmium tetroxide, dehydrated using increasing concentrations of ethanol (30–100%), then subjected to drying at the critical point with carbon dioxide, metalized with gold, and analyzed in a high-resolution double-beam electron microscope (FEI SCIOS, Oregon, USA).

Transmission electron microscopy (TEM) was used to detect ultrastructural changes. The parasites and infected cells were fixed with 2.5% glutaraldehyde in 0.1 M sodium cacodylate buffer, post-fixed in 1% osmium tetroxide solution and 0.8% potassium ferrocyanide at room temperature and protected from light. Afterwards, the cells were dehydrated using increasing concentrations of acetone (50–100%), followed by adding EPON™ resin, and polymerized in an oven at 60°C for 72 h. Ultrathin sections were made on an ultramicrotome (PowerTomer BMC – Germany), deposited on a copper grid, and contrasted with uranyl acetate (5%) and lead citrate (2%) for 20 and 10 min, respectively. TEM analysis was performed on a JEOL JEM 1400 instrument (Tokyo, Japan).

### 2.9 Determination of cell membrane integrity

Promastigote forms (10^6^) treated with OEO (IC_50_ and 2× IC_50_) for 24 h were incubated with propidium iodide (PI; Sigma; 0.5 μg/mL) for 15 min at 25°C. Parasites were analyzed using a fluorescence microplate reader (Victor X3, PerkinElmer) with an excitation wavelength of 480 nm and emission of 580 nm. The obtained fluorescence values were normalized by the number of parasites.

### 2.10 Determination of phosphatidylserine exposure

Phosphatidylserine exposure was detected using Annexin-V FITC (Invitrogen, Eugene, OR). Promastigote forms (10^6^) treated with OEO (IC_50_ and 2× IC_50_) for 24h were incubated with 5 μL of Annexin-V FITC for 15 min at 25°C. Camptothecin (Sigma; 10 μM) was used as a positive control. The data were obtained from a fluorescence microplate reader (Victor X3, PerkinElmer) at an excitation wavelength of 488 nm and emission of 520 nm. The obtained fluorescence values were normalized by the number of parasites.

### 2.11 Mammalian cell culture

THP-1 cell lines (ATCC^®^TIB-202™), monocyte culture derived from the peripheral blood of a patient with acute monocytic leukemia, were used for the experiment. Cells were cultured in RPMI 1640 medium supplemented with 1% L-glutamine, 10% sodium bicarbonate, 5% FBS, 10 u/mL penicillin, and 10 µg/mL streptomycin (GIBCO, Invitrogen). Cells were maintained in 75 cm^2^ culture flasks in an incubator (37° C, 5% CO2), and were subcultured every 7 days.

### 2.12 Cell viability

THP-1 cells (5 × 10^4^ cells/ml) were cultured in 96-well plates with 200 μL of RPMI 1640 medium (10% SFB) + 0.5% PMA (phorbol-12-myristate-13- acetate) for 48 h (37°C, 5% CO2), for differentiation into macrophages. Cells were then washed, added to RPMI 1640 medium (10% SFB) and left resting for 72 h. Next, adherent macrophages were incubated with OEO (6.25, 12.5, 25, 50, 100 and 150 μg/ml) and cultured for 24 h under the same conditions. The cells were then washed with PBS, and 3-(4,5-dimethylthiazol-2-yl)-2,5- diphenyltetrazolium bromide (MTT) (Sigma-Aldrich, St. Louis, MO, USA) (5 mg/ml) was added to the cells and incubated for 4 h. DMSO 0.01% was used as vehicle and cells containing only RPMI medium were used as negative control.

### 2.13 Anti-amastigote assay

THP-1 monocytes (1 x 10^5^) were plated, then incubated with PMA 0.5% for differentiation into macrophages. After 48 h, these cells were washed 2x with sterile PBS and maintained in RPMI 1640 medium (10% SFB). 1 x 10^6^ promastigotes of *L. infantum* (MCAN/BR/97/p142 and MS strains) were then added to the cells for 24 h, and subsequently, treated with OEO at concentrations of 25, 50, 75 µg/mL for 24 h. AmB was used as a positive control. Reading was performed on a BD AccuriTM- C6 Plus flow cytometer (BD Biosciences, San Jose, CA, USA).

### 2.14 Statistical analysis

Three independent experiments were performed. Statistical differences were determined by analysis of variance (ANOVA), followed by the Tukey test for multiple comparisons using GraphPad Prism 6.01 for Windows (GraphPad Software, San Diego CA). Data was expressed as mean ± SEM and differences were considered significant when P-value ≤ 0.05. The values were also categorized by: ∗ (*p* < 0.05); ∗∗ (*p* < 0.01); ∗∗∗ (*p* < 0.001); ∗∗∗∗ (*p* < 0.0001).

## 3 Results

**Figure 1:**
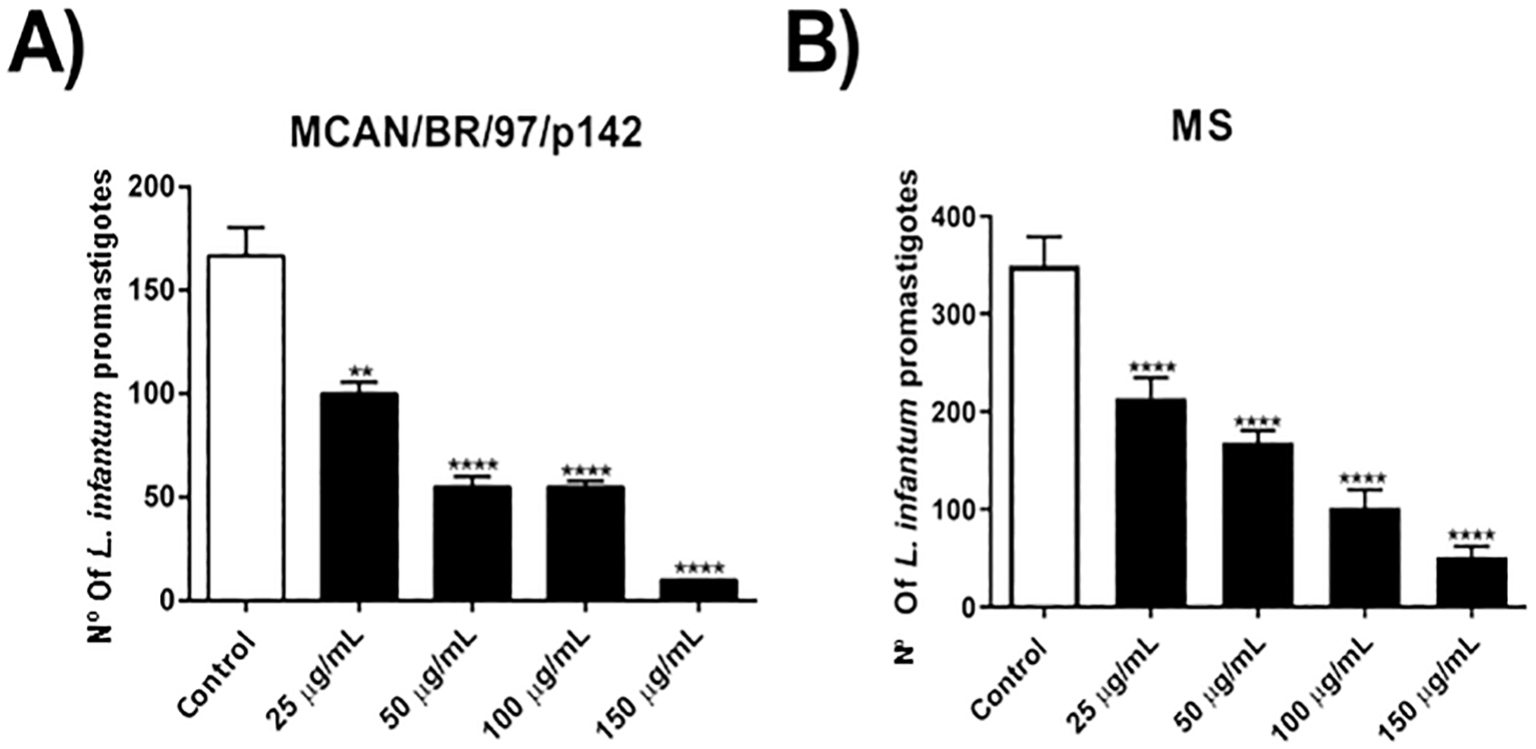
Treatment with OEO has activity on promastigote forms of *Leishmania infantum* strains. **(A, B)** 1 x 10^6^ parasites were incubated with different concentrations of OEO (25, 50, 100 and 150 µg/mL) for 24 h and counted in a Neubauer chamber. The values shown are expressed as mean ± standard error. **p < 0.01; ***p < 0.001; ****p < 0.0001. The experiment was carried out in triplicate and repeated five times.

At first, we evaluated the direct activity of OEO on promastigote forms of the MCAN/BR/97/p142 and MS strains of *Leishmania infantum*. It was verified that all the concentrations tested significantly reduced the viability of the parasites of both strains (**Figures 1A–C**). The IC5O and 2x IC_50_ were of 43.61 and 87.22 μg/mL for the MS isolate, respectively. These values were relatively high compared to the reference strain MCAN/BR/97/p142, which IC_50_ was of 12.53 and 2x IC_50_ was of 25.06 μg/mL.

**Figure 2:**
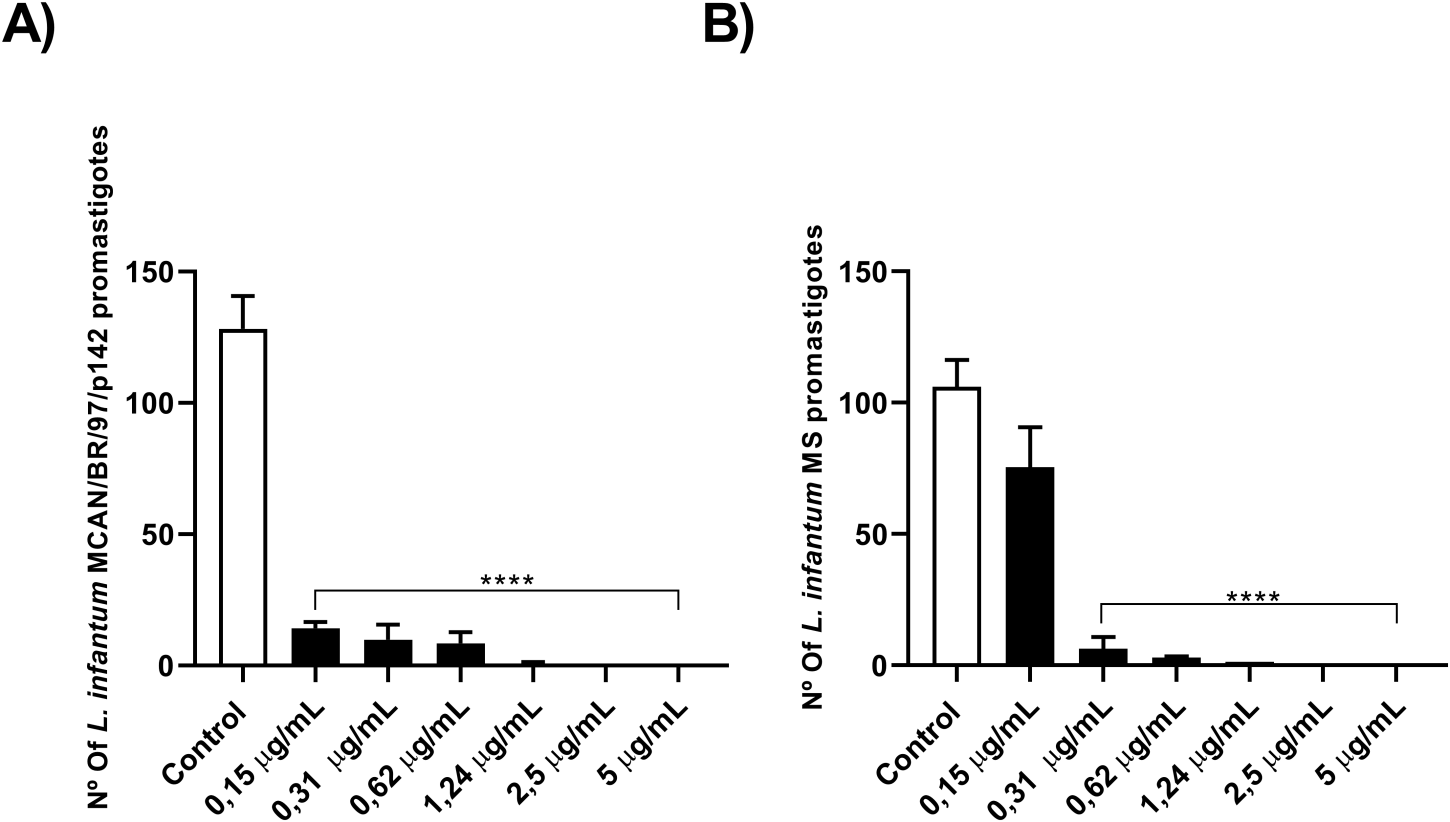
The natural infection strain demonstrated greater resistance to AmB. **(A, B)** 1 x 10^6^ parasites were incubated with different concentrations of AmB (0.15; 0.31; 0.62; 1.24; 2.5 and 5 µg/mL) for 24 h and counted in a Neubauer chamber. Values are expressed as mean ± standard error. **p < 0.01; ***p < 0.001; ****p < 0.0001. The experiment was carried out in triplicate and repeated five times.

To check whether the isolated strain was indeed more resistant, or whether they were only resistant to OEO, we incubated promastigote forms of both strains and treated them with different concentrations of Amphotericin B (AmB). As observed in **Figures 2A, B**, the MS strain was less susceptible to AmB compared to the MCAN/BR/97/p142 strain, with IC_50_ values of 0.2126 and 0.1453 μg/mL, respectively. This pattern of greater susceptibility of the commercial strain was similar to that obtained in the treatment with OEO.

**Figure 3:**
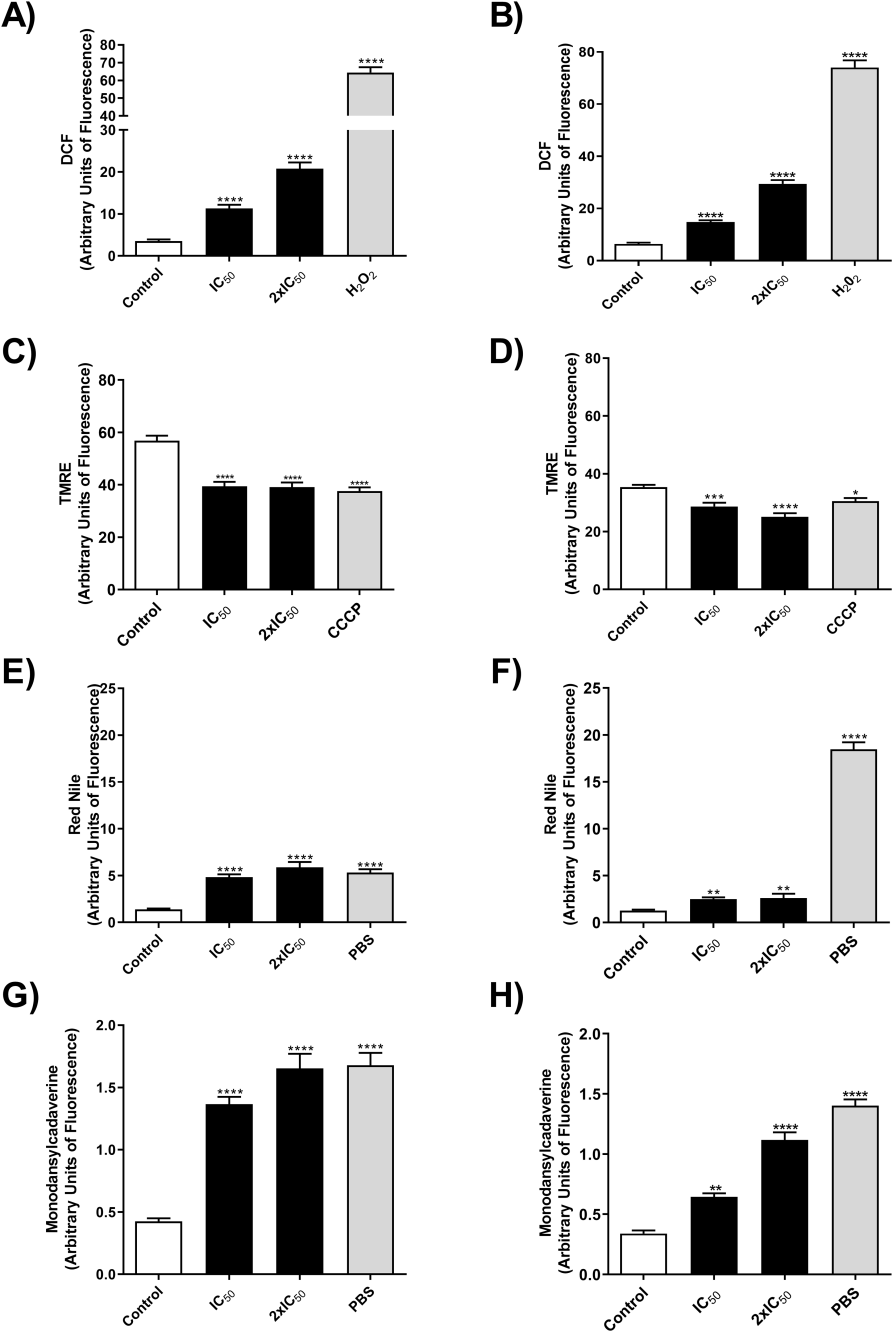
OEO increases the production of ROS, leads to mitochondrial depolarization, formation of lipid droplets and an increase in autophagic vacuoles in promastigote forms of *L. infantum* strains. **(A, B)** MCAN/BR/97/p142 and MS strains ROS production by DCF fluorescence, respectively. **(C, D)** MCAN/BR/97/p142 and MS strains alteration of ΔΨmby TMRE, respectively. **(E, F)** MCAN/BR/97/p142 and MS strains, accumulation of cytoplasmic LD by Nile Red, respectively, and **(G, H)** MCAN/BR/97/p142 and MS strains, accumulation of cytoplasmic autophagic vacuoles, respectively. Promastigotes were cultivated in medium 199 were used as a negative control, H2O2, CCCP and PBS as a positive control. The values shown are expressed as mean ± standard deviation.

**Figure 4:**
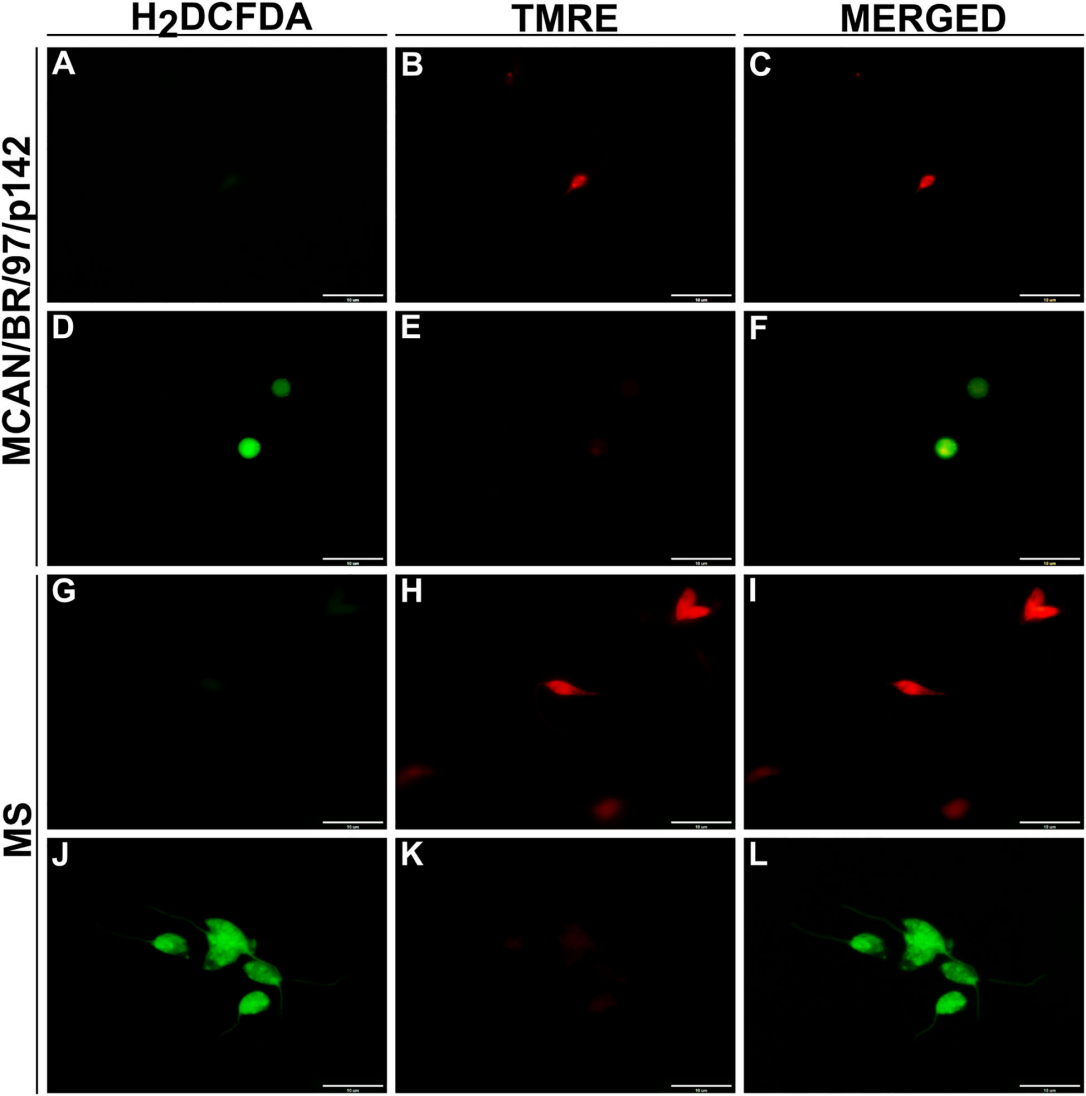
OEO’s effect on reactive oxygen species production and mitochondrial membrane integrity in *L. infantum* promastigotes. Promastigotes were treated for 24 hours with IC_50_ of OEO. Representative images taken by fluorescence microscopy show ROS levels by DCF fluorescence (green), a marker for total reactive oxygen species; and mitochondrial membrane by the TMRE marking (red), a marker for mitochondrial membrane potential intensity. Control (untreated parasites). MCAN/BR/97/p142 strain: **(A–C)** control, **(D–F)** treated with OEO’s IC_50_; MS strain: **(G–I)** control, **(J–L)** treated with OEO’s IC_50_. Scale bars = 10 μm. The data represent the mean ± SEM of three independent experiments performed in duplicate.

Knowing that OEO has direct activity on promastigote forms of *L. infantum*, we investigated the mechanisms of action involved in killing the parasite. To this end, we evaluated the production of reactive oxygen species (ROS), mitochondrial membrane potential, the presence of lipid bodies and autophagic vacuoles. As a result, treatment with OEO was able to significantly increase ROS production in promastigote forms (**Figures 3A, B**), lead to mitochondrial depolarization (**Figures 3C, D**), as well as the accumulation of cytoplasmic lipid droplets (**Figures 3E, F**) and the formation of autophagic vacuoles (**Figures 3G, H**) in both *L. infantum *strains. Fluorescence microscopy images corroborate the aforementioned data, showing an increase in ROS in treated promastigotes and a decrease in mitochondrial membrane potential (**Figure 4**).

Promastigotes (10^6^) of the MCAN/BR/97/p142 and MS strains were treated with OEO (IC_50_ and 2× IC_50_) for 24h and analyzed by fluorescence intensity for the following parameters: ROS production by DCF fluorescence in the reference strain (A) and MS strain (B), changes in mitochondrial membrane potential (ΔΨm) by TMRE in the reference (C) and MS strain (D), accumulation of cytoplasmic lipid droplets by Nile Red staining in the reference (E) and MS strain (F,) and formation of autophagic vacuoles by monodansylcadaverine in the reference (G) and MS strain (H). Promastigote forms grown in Medium 199 were used as a negative control, while H_2_O_2_, CCCP and PBS, as positive controls. The values shown are expressed as mean ± standard error. * p < 0.05; ** p < 0.01; *** p < 0.001 and **** p < 0.0001.

**Figure 5:**
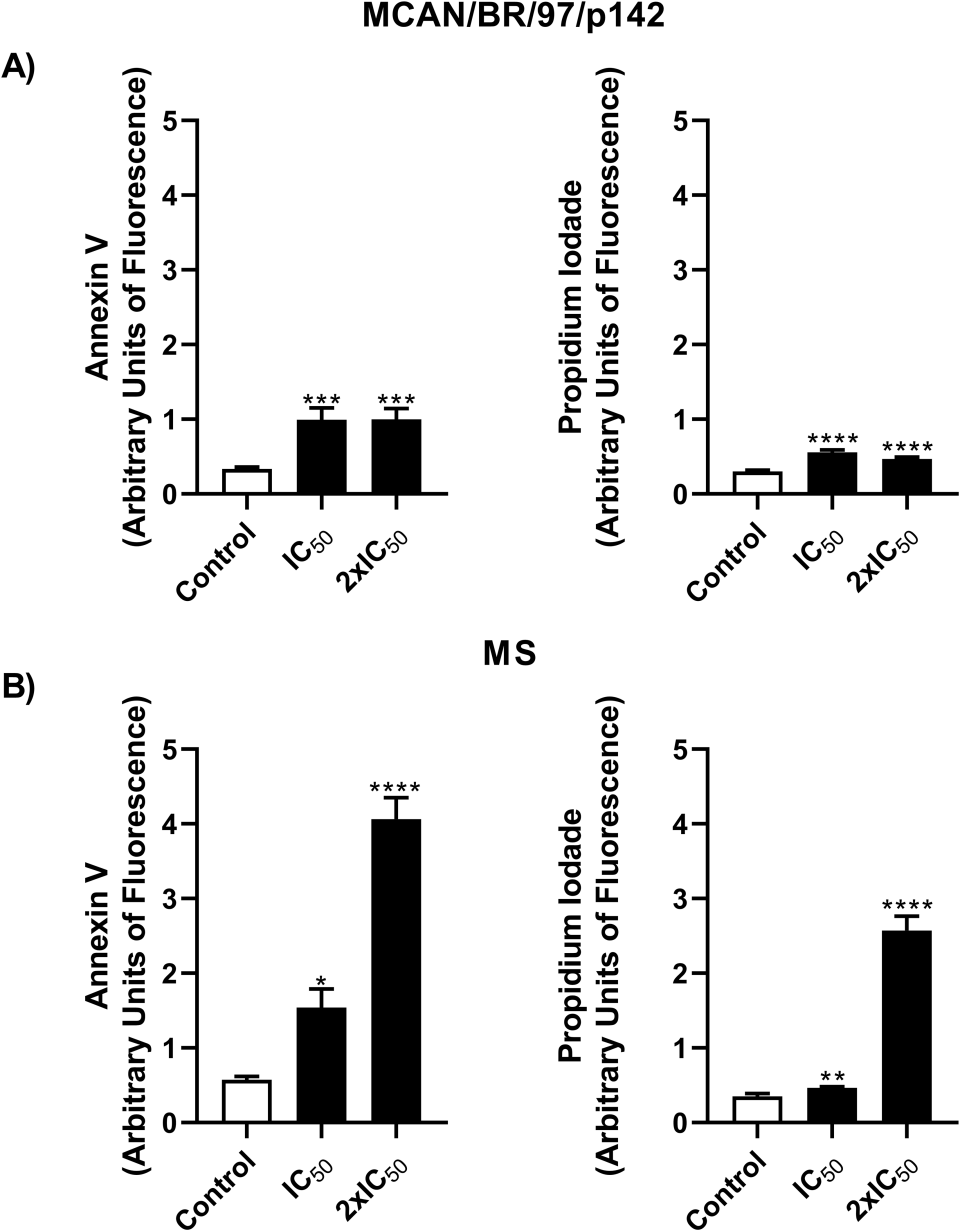
Treatment with OEO leads to the death of *Leishmania infantum* via a apoptosis-like pathway. Promastigotes (10^6^) of the MCAN/BR/97/p142 and MS strains were treated with OEO (IC_50_ and 2 × IC_50_) for 24 h and labeled with Annexin V for phosphatidylserine exposure and PI for cell membrane integrity analysis. **(A)** MCAN/BR/97/p142 strain; **(B)** MS strain. Medium 199 was used as a negative control. Values are expressed as mean ± standard error. *p < 0.05; **p < 0.01 and ****p < 0.0001.

In light of the cellular stress induced by the treatment with OEO, we evaluated next whether these mechanisms would effectively lead to the death of the parasite through known pathways such as apoptosis or necrosis. **Figure 5** shows a proportional increase in the fluorescence of Annexin-V and PI when compared to the control, which allows us to infer that the parasites are dying by apoptosis. This effect was observed in both strains.

**Figure 6:**
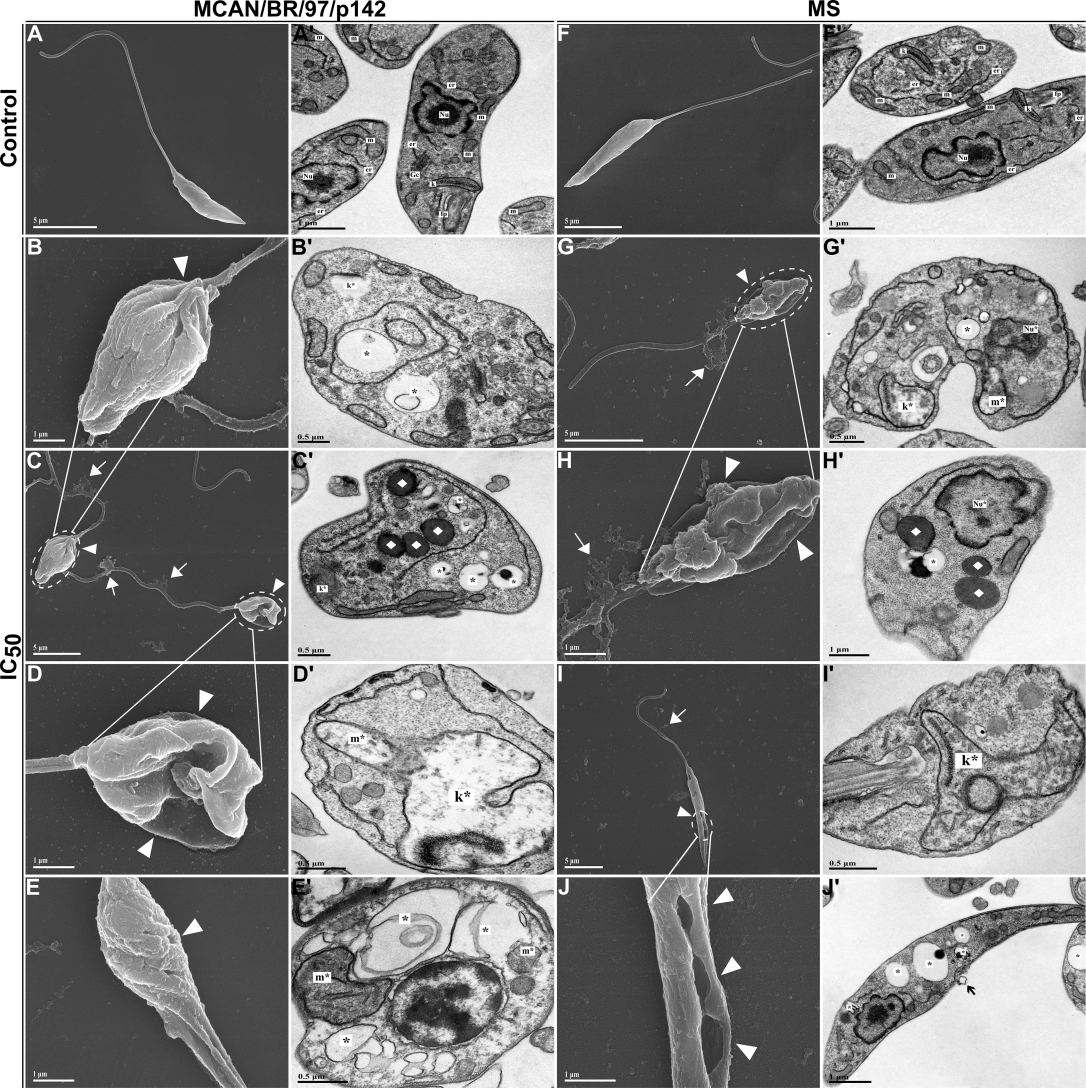
Morphological and ultrastructural changes in promastigote forms of *Leishmania infantum* treated with IC_50_ of OEO for 24 h **(A–J)** Scanning electron microscopy. **(A’-J’)** Transmission electron microscopy. **(A, A’)** Promastigotes of *L. infantum* strain MCAN/BR/97/p142 untreated; **(B–E, B’-E’)** Promastigotes of *L. infantum* strain MCAN/BR/97/p142 treated with IC_50_ of OEO; **(F, F’)** Promastigotes of *L. infantum* strain MS untreated; **(G–J, G’-J’)** Promastigotes of *L. infantum* strain MS treated with IC_50_ of OEO. Flagellar pocket (fp); Kinetoplast (k); Golgi complex (Gc); Mitochondrion (m); Nucleus (Nu); Endoplasmic reticulum (er); Changes in the kinetoplast region (k*); Mitochondrial changes (m*); Nuclear changes (Nu*); Extravasation of cytoplasmic content (◻white); Damage to the plasma membrane (▸white); Membrane blebs (◻black); Autophagic vacuoles (*); Lipid bodies (♦). Scale bars: 5 µm **(A, A’, C, F, F’, G, H’, I, J’)**, 1 µm **(B, D, E, H, J)**, 0.5 µm **(B’-E’, G’, I’)**.

Next, we analyzed whether the treatment with OEO caused morphological and ultrastructural changes in promastigote forms of the *L. infantum* strains, which were analyzed using scanning electron microscopy (SEM) and transmission electron microscopy (TEM) (**Figure 6**). Typical untreated promastigotes (control) show an elongated body, smooth cell membrane and preserved flagella as pictured in **Figures 6A, F**. However, when treated for 24 h with the IC_50_ of OEO, the parasites exhibited a rounded shape, reduced body size, roughness on the cell surface and apparent leakage of cytoplasmic content. (**Figures 6B–E, G–J**). Additionally, the TEM images show mitochondrial and kinetoplast swelling and nuclear disorganization; as well as, an increase in lipid bodies and autophagic vacuoles (**Figure 6B’, C’–E’, G’–J’**). This contrasts with the cellular organization of the untreated promastigote (**Figures 6A’, F’**).

**Figure 7:**
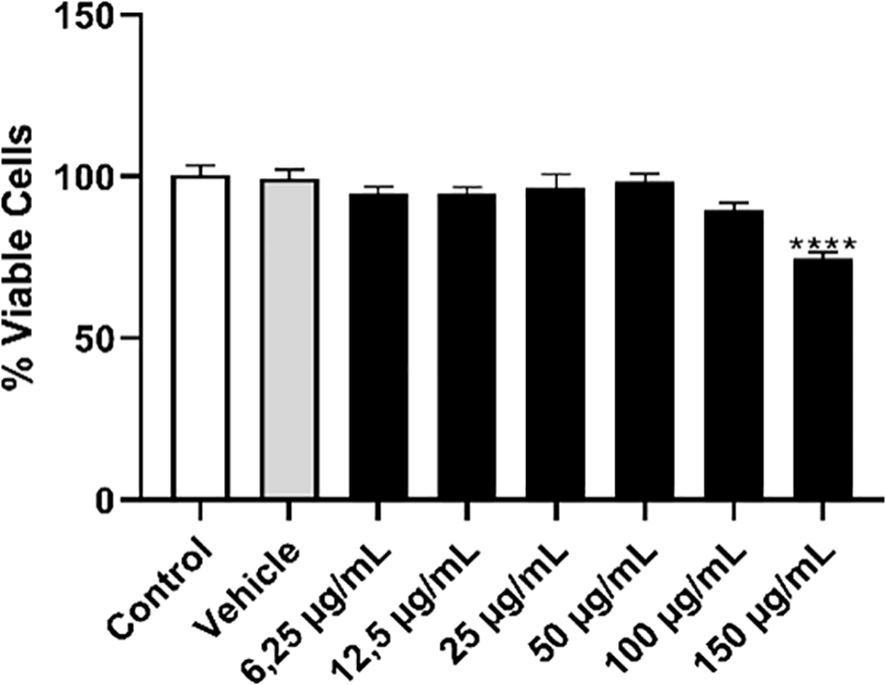
Treatment with OEO showed low toxicity in the THP-1 monocytic cell line. THP-1 monocytes (5 x 10^4^) were plated and incubated with PMA for differentiation into macrophages. After 48 h, these cells were washed, cultured in RPMI medium, and treated with different concentrations of OEO (6.25- 150 µg/mL). DMSO 0.2% was used as the vehicle. The values shown are expressed as mean ± standard deviation. ****p < 0,001.

Knowing the effect of OEO on promastigote forms, we investigated its potential on L. infantum amastigotes internalized in THP-1 macrophages. Initially, we assessed whether OEO was toxic to the THP-1 cell line. As a result, we observed that the treatment caused cytotoxicity (**Figure 7**) only at the highest concentration of 150 µg/mL, showing a 25.89% (± 1.995) reduction in cell viability; thus, this concentration was not used in subsequent experiments.

**Figure 8:**
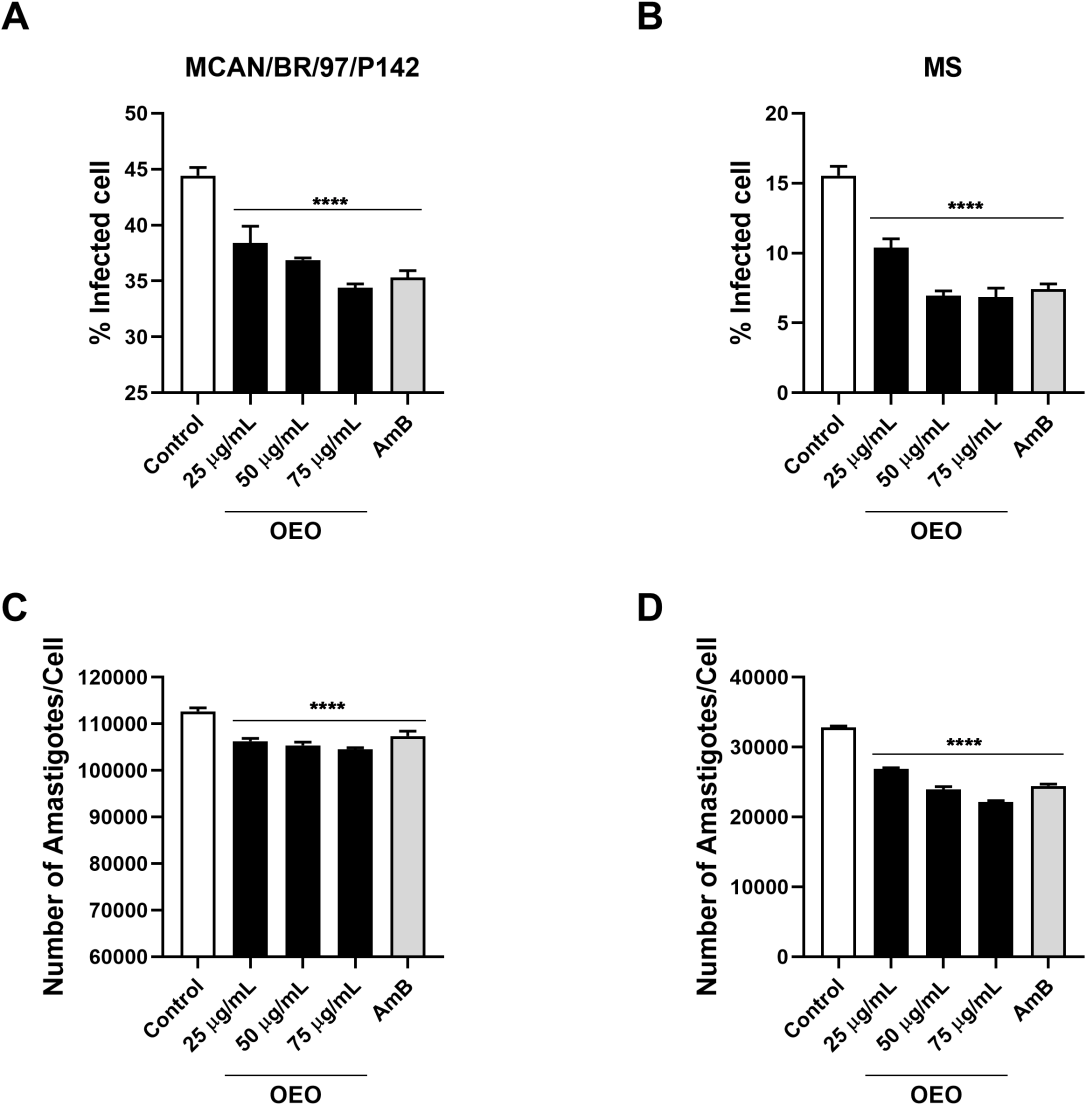
Treatment with OEO reduces the number of infected macrophages as well as the number of amastigotes per macrophage. THP-1 monocytes (10^5^) were plated and then incubated with PMA for differentiation into macrophages. After 48 h, these cells were washed, added to RPMI medium, and infected with 5x10^6^ promastigotes of *Leishmania infantum* (**A, C**: strain MCAN/BR/97/p142; **B, D**: strain MS). After 24 h, the strains were treated with the concentrations 25, 50, 75 µg/mL of OEO. The percentage of infected cells **(A, B)** and the number of amastigotes per macrophage **(C, D)** were evaluated. ****p<0,001.

Since the treatment showed no significant cellular toxicity, we evaluated its activity on the intramacrophage forms of the parasite. Our results showed that treatment with OEO was able to significantly reduce the percentage of cells infected with the MCAN/BR/97/p142 and MS strain, as well as the number of amastigotes per macrophage at all the concentrations tested. (**Figure 8**). It was also observed that the rate of macrophages infected by the MS strain was considerably lower than those infected by the reference strain; however, the number of parasites per cell was higher in macrophages infected with *L. infantum* MS.

**Figure 9:**
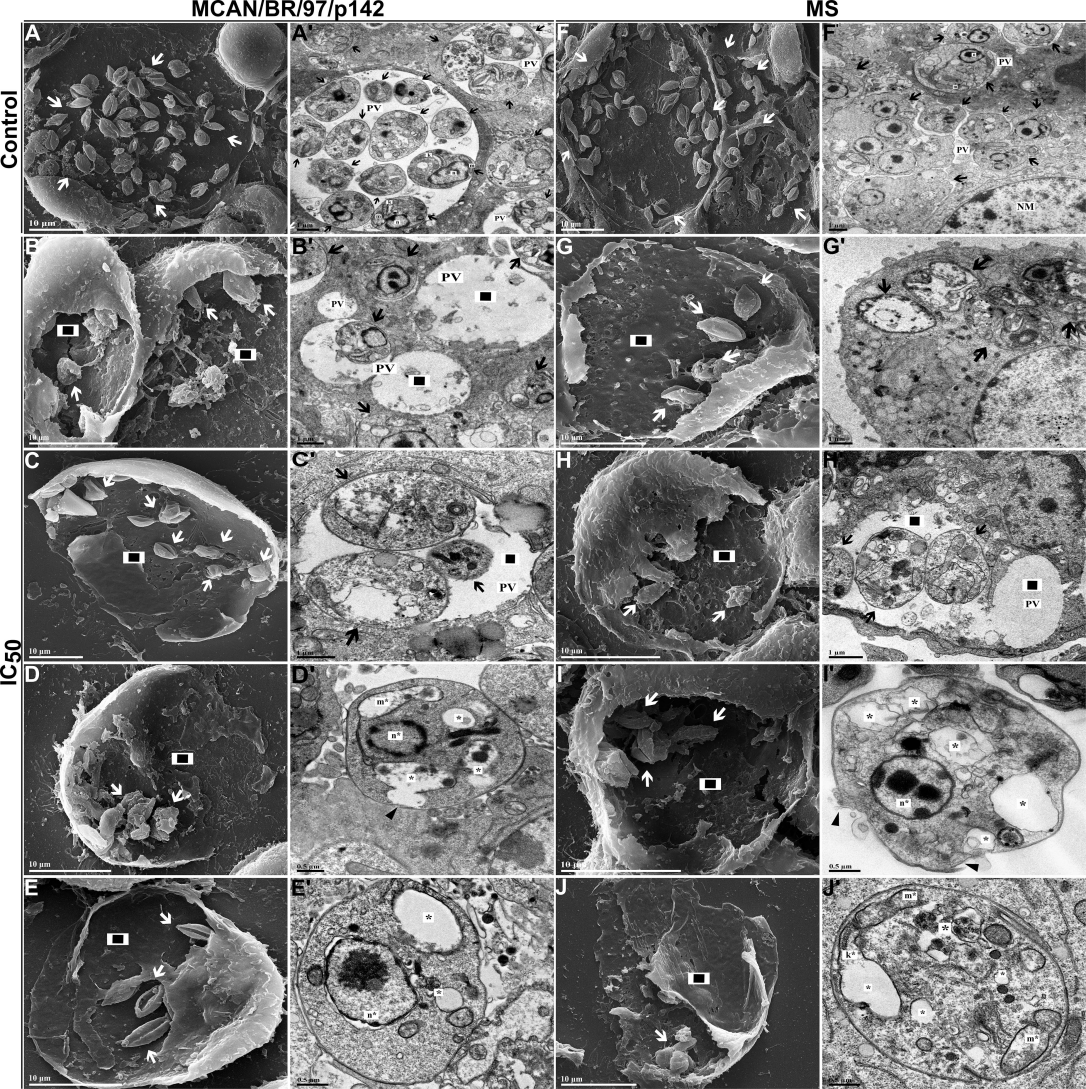
Morphological and ultrastructural changes in amastigote forms of *Leishmania infantum* treated with IC_50_ of OEO for 24 h. **(A–J)** Scanning electron microscopy. **(A’-J’)** Transmission electron microscopy. **(A, A’)** Amastigotes of *L. infantum* strain MCAN/BR/97/p142 untreated; **(B–E, B’-E’)** Amastigotes of *L. infantum* strain MCAN/BR/97/p142 treated with IC_50_ of OEO; **(F, F’)** amastigotes of *L. infantum* strain MS untreated; **(G–J, G’-J’)** Amastigotes of *L. infantum* strain MS treated with IC_50_ of OEO. Kinetoplast (k); Mitochondrion (m); Nucleus of amastigote (n); Nucleus of macrophage (NM); Parasitophorous vacuole (PV); Alteration in the kinetoplast region (k*); Mitochondrial changes (m*); Nuclear alterations (n*); (◻black/white) Damage to the plasma membrane. (▸black); Reduction in the amount of amastigotes (◼); Autophagic vacuoles (*). Scale bars: 10 µm **(A–J)**, 1 µm **(A’-C’, F’-H’)**, 0.5 µm **(D’, E’, I’, J’)**.

To corroborate our data, both *L. infantum* strains were plated and treated with OEO IC50 and subjected to TEM and SEM. We observed a significant reduction in the number of intramacrophagic amastigotes, which displayed plasma membrane damage, nuclear alterations, changes in mitochondria and the kinetoplast region when treated (**Figures 9B, B’, C, C’, D, D’, E, E’, G, G’, H, H’, I, I’, J, J’**) when compared to untreated amastigotes (**Figures 9A- A’, F, F’**).

## 4 Discussion

The conventional treatment used for leishmaniasis consists of parenteral administration, and although it has good efficacy, it has toxic side effects which can lead the patient to discontinue treatment. As a result, resistant strains have emerged in recent years, limiting the efficacy of the treatment in several countries (Anversa et al., 2018; Torres-Guerrero et al., 2017).

Given the need for new therapies, several studies have reported the activity of the main compounds of oregano essential oil (OEO) against promastigote forms of different *Leishmania* species, (Morales-Yuste et al., 2010) showed that carvacrol was the compound with the highest activity against promastigote forms of *L. infantum*. On the other hand, authors have shown that thymol was the most effective compound against the promastigote forms of *L. amazonensis* and *L. Infantum* (de Medeiros et al., 2011; Youssefi et al., 2019). However, significant interactions were observed among the various components of OEO, suggesting that its maximum effectiveness is achieved through the combination of its constituents rather than as isolated compounds (Bassolé and Juliani, 2012).

In the current study, we demonstrated that OEO showed activity against two different strains of *L. infantum*: one from a naturally infected dog (MS) and a reference strain (MCAN/BR/97/p142). In addition, it was demonstrated that the dog-isolated strain showed greater resistance when compared to the reference strain, indicated by higher IC_50_ values; this corroborates previous works in which strains isolated from naturally infected animals exhibited resistance (Maia et al., 2013), including cross-resistance between commonly used drugs (Gonçalves et al., 2021)

Reactive oxygen species (ROS) are considered important signaling molecules that regulate essential components of cell function, making them potentially antimicrobial (Dryden, 2018; Kumari et al., 2017). High ROS production is directly linked to lipid peroxidation, a process in which free oxygen radicals attack polyunsaturated fatty acids (PUFAs) present in cell membranes. This attack results in the formation of lipid peroxides, which, if not effectively neutralized, compromise the structure and function of both cellular and mitochondrial membranes (Su et al., 2019). Lipid peroxidation, by affecting membranes, can induce the formation of additional free radicals, leading to a chain of reactions that propagate cellular damage, such as the loss of critical components of the electron transport chain and mitochondrial dysfunction (Sinha et al., 2013), ultimately leading to positive feedback and resulting in programmed cell death, such as apoptosis (Zheng et al., 2024).

Authors have highlighted the relevance of this process in parasites such as *Leishmania*, where the interaction between ROS and PUFAs contributes to mitochondrial dysfunction, mitochondrial membrane depolarization, and cytochrome c release, activating caspases and promoting apoptosis (Basmaciyan et al., 2018). In light of this, our work demonstrated that treatment with IC50 and 2x IC50 concentrations of oregano essential oil (OEO) significantly increased ROS levels in promastigote forms, both in the reference and isolated strains, suggesting that the parasite was undergoing cellular stress. Furthermore, corroborating our TEM and SEM data, studies show that increased ROS levels can lead to mitochondrial membrane destruction through PUFA oxidation, causing mitochondrial membrane swelling, resulting in the irreversible opening of the mitochondrial permeability transition pore and mitochondrial membrane permeability collapse (Wang et al., 2023).

Due to the oxidative stress induced by treatment with OEO, the parasite also exhibited mitochondrial depolarization. The mitochondrion is an organelle of great importance for cell survival, providing energy by generating ATP through oxidative phosphorylation (Saudagar and Dubey, 2014). *Leishmania* parasites have a single mitochondrion, and the proper functioning of this organelle is essential to ensure parasite survival (Shadab et al., 2017). In this regard, mitochondrial dysfunctions can favor the formation of lipid droplets, composed mainly of neutral lipids, triglycerides, and sterols, which can alter metabolic pathways of glycolysis and fatty acid biosynthesis in stressful situations (Lee et al., 2013).

This series of cellular alterations and the accumulation of autophagic vacuoles observed in parasites treated with OEO leads us to believe that the cell is attempting recovery, with autophagy serving as a physiological pathway involved in the removal of damaged cellular components and associated with cell cycling, survival, or even cell death when overactivated (Dolai and Adak, 2014; Machado et al., 2017). Recent studies suggest that the interaction between ROS and autophagy in parasites such as *Leishmania* may be mediated by mechanisms such as metacaspase, an enzyme that, although absent in mammals, performs a similar role to caspases in ancestral eukaryotes, regulating both apoptosis and autophagy. When oxidative stress is excessive, autophagy may become overloaded, leading to the induction of apoptosis (Basmaciyan et al., 2018).

As previously mentioned, products generated by oxidative stress, derived from lipid peroxidation, may interact with membrane receptors to induce apoptosis in various ways, primarily through the peroxidation of cardiolipin, a phospholipid of the inner mitochondrial membrane (Su et al., 2019; Zheng et al., 2024). Apoptosis is a death process that occurs frequently in these parasites, involving physical and biochemical changes such as cellular stress, mitochondrial damage, and morphological alterations, such as body shrinkage, phosphatidylserine exposure, and nuclear fragmentation (Basmaciyan et al., 2018). Similar results were found by Bortoleti et al. (2019), Miranda-Sapla et al. (2019) and Tomiotto-Pellissier et al. (2022). In line with this, our assays identified an increase in both cell death markers when treated with IC50 of OEO (12.53 µg/mL for the reference strain; 43.61 µg/mL, isolated strain), exhibiting phosphatidylserine exposure and membrane permeabilization; this corroborates the SEM data, where both strains showed shrinkage and rounding of the cell body. In addition, membrane roughness and cytoplasmic content extravasation were also identified, characteristics commonly associated with death by late apoptosis (Basmaciyan and Casanova, 2019; Poon et al., 2010). This may be due to the highly lipophilic nature of OEO, which allows its easy absorption by the cell membrane, destabilizing the phospholipid bilayer, altering the permeability of membranes, including the mitochondrial membrane, and thus resulting in apoptosis (Wink, 2008).

Given that *Leishmania* is an intracellular parasite, it is of great importance to evaluate the effects of OEO on amastigote forms of the parasite, as well as on mammalian host cells, ensuring it does not induce cytotoxic activity (Sundar and Singh, 2018).Thus, our results revealed that OEO was not toxic to THP-1 macrophages, except at the highest concentration (150 µg/mL). This corroborates the work of Tomiotto-Pellissier et al. (2022), which demonstrated that concentrations up to 100 µg/mL of OEO showed no toxicity to peritoneal macrophages.

Other than displaying low cytotoxicity, all concentrations of OEO were able to reduce the rate of infected macrophages and number of amastigotes per macrophage in a similar way as AmB. SEM and TEM images corroborate these findings, illustrating the damage to the parasite membrane and cellular stress components after receival of treatment. Similar results have been reported in other studies that demonstrated that the elimination of intracellular forms of *L. amazonensis* after treatment with OEO can occur through the disruption of the parasite’s cellular metabolism and the formation of autophagic vacuoles (Alves et al., 2023; Tomiotto-Pellissier et al., 2022).

Compared to the reference strain, the MS strain exhibited greater resistance to treatment, indicated by a higher IC_50_ value, and therefore required a higher dose of OEO to activate the same death pathways as the reference strain. However, even in the MS strain, our results demonstrated that treatment with OEO for 24h induced cellular stress, triggering an apoptosis-like process in promastigote forms. Yet, the infection rate of the dog-isolated strain (MS) was considerably lower than that of the reference strain. Differences in the infectivity of strains have already been shown, and can occur even when the strains are in their respective stationary phases (Palacios et al., 2017). This factor can be supported by the fact that the greater the virulence *in vivo* of the strain, the lower is the infectivity of promastigotes *in vitro* (Resende et al., 2020). Despite this, OEO displayed promising results in amastigote forms of the parasite, significantly reducing the rate of infection and the number of parasites per infected cell.

## 5 Conclusion

This research showed that OEO has activity against different strains of *L. infantum*, including one isolated from a naturally infected dog. The dog-isolated strain exhibited greater resistance compared to the reference strain, which is consistent with previous studies. Treatment with OEO increased cellular stress levels, leading to parasite death, likely through the activation of apoptosis pathways. In intracellular amastigotes assays, OEO significantly reduced the rate of infected macrophages and the number of parasites per cell, similarly to treatment with AmB. Even in the dog-isolated resistant strain, OEO demonstrated high efficacy in reducing parasite load, highlighting its anti-*Leishmania* potential *in vitro*. However, further studies are needed to better elucidate the behavior of OEO in the treatment of human and canine VL.

## Data Availability

The datasets presented in this article are not readily available because The raw data is restricted due to the intellectual property of the authors, so if any of the reviewers have any doubts, they can ask the authors. Requests to access the datasets should be directed to Ana Carolina Jacob Rodrigues carolinajacob.ana@gmail.com.
